# Role of Smac/DIABLO in cancer progression

**DOI:** 10.1186/1756-9966-27-48

**Published:** 2008-09-26

**Authors:** Gustavo Martinez-Ruiz, Vilma Maldonado, Gisela Ceballos-Cancino, Juan P Reyes Grajeda, Jorge Melendez-Zajgla

**Affiliations:** 1 Functional Cancer Genomics Laboratory, National Institute of Genomic Medicine, Periferico Sur 4124, Torre Zafiro II 5to piso, Col. Ex-Rancho de Anzaldo, Alvaro Obregon 01900, Mexico City, México; 2 Molecular Biology Laboratory, Subdireccion de Investigacion Basica, Instituto Nacional de Cancerologia, Mexico City, 14000, México; 3 Medical Proteomics unit, National Institute of Genomic Medicine, Mexico City, 01900, México

## Abstract

Second mitochondria-derived activator of caspase/direct inhibitor of apoptosis-binding protein with low pI (Smac/DIABLO) is a proapoptogenic mitochondrial protein that is released to the cytosol in response to diverse apoptotic stimuli, including commonly used chemotherapeutic drugs. In the cytosol, Smac/DIABLO interacts and antagonizes inhibitors of apoptosis proteins (IAPs), thus allowing the activation of caspases and apoptosis. This activity has prompted the synthesis of peptidomimetics that could potentially be used in cancer therapy. For these reasons, several authors have analyzed the expression levels of Smac/DIABLO in samples of patients from different tumors. Although dissimilar results have been found, a tissue-specific role of this protein emerges from the data. The objective of this review is to present the current knowledge of the Smac/DIABLO role in cancer and its possible use as a marker or therapeutic target for drug design.

## Background

Cancer cells share six features that distinguish them from normal cells: Autocrine production of growth signals, inability to respond to anti-growth signals, sustained angiogenesis, limitless replicative potential, tissue invasion and metastasis, and apoptosis avoidance [1]. This type of cell death is fundamental for the maintenance of tissue homeostasis and immune system development [2]. Tumor cells are subjected to stressful internal and external environments, but nevertheless are resistant to apoptosis.

Apoptosis can be activated through two pathways: The extrinsic pathway (mediated by death receptors) or the intrinsic pathway (mediated by mitochondria). The former is activated in response to the engagement of ligands such as CD95 or TNF-α with their receptors. This in turn induces the recruitment of adapter proteins (FADD, TRADD o RAIDD) to form the so-called death-inducing signal complex (DISC), which activates caspase-8. In turn, caspase 8 activates effector caspases by catalytic cleavage. The intrinsic pathway is induced by several different stimuli like antineoplastic drugs, hypoxia, irradiation, growth factor withdrawal and heat shock. These stimuli provoke the mitochondrial outer membrane permeabilization (MOMP) and the release of proteins from the intermembrane space, such as cytochrome-c, Smac/DIABLO, Omi/HtrA2 and AIF to the cytosol [3]. This release allows the assemble of a multiprotein complex, the apoptosome, that includes cytochrome-c, procaspase-9, dATP and cytosolic apoptosis inductor factor-1 (Apaf-1) [4]. The apoptosome activates caspase-9, which in turn induces the activation of effector caspases-3, -6 and -7 [5]. The effector caspases cleave their cellular specific substrates and generate the typical morphology of apoptosis.

The activity of mature caspases is negatively regulated by their interaction with inhibitor of apoptosis proteins (IAPs) [6,7]. This protein family is comprised by X-linked inhibitor of apoptosis (XIAP), cellular IAP-1 (c-IAP1), cellular IAP-2 (c-IAP2), Testis specific IAP (Ts-IAP), survivin, livin and BRUCE/Apollon [8]. The more studied member is XIAP, formed by three BIR (Baculoviral IAP Repeat) domains located in the NH_2_-terminus and one RING (Really Interesting New Gene) domain in the CO_2_H-terminus. The linker region between the BIR1 and BIR2 is implicated in the inhibition of caspase-3 and -7 whereas the BIR2 domain inhibits caspase-7 in a non-competitive manner [9]. Caspase-9 activity is inhibited by its association with the BIR3 domain of XIAP [10]. In addition, it has been determined that the RING domain of XIAP has E3 ubiquitin ligase activity toward caspases, provoking their degradation by the proteasome after their interaction [11,12].

Smac (Second mitochondria-derived activator of caspase) protein, also known as DIABLO (Direct Inhibitor of Apoptosis-Binding protein with LOw pI), is codified by a nuclear gene. Its protein presents an NH_2_-terminus that serves as mitochondrial targeting signal (MTS). The mature form of Smac/DIABLO is originated by the cleavage of this signal. In the presence of apoptotic stimuli, mature Smac/DIABLO is release to the cytosol [13]. There, Smac/DIABLO has a pro-apoptotic effect that is mediated by its interaction with IAPs and the release of caspases from them. Structural data had established that Smac/DIABLO requires to form homodimers to interact with IAPs [14]. A particular NH_2_- terminal motif, consisting of four amino acids, Ala-Val-Pro-Ile, is responsible for the interaction with IAPs [14,15]. It has been demonstrated that Smac/DIABLO interacts with the BIR2 and BIR3 domains of XIAP, allowing the release of caspase-3 [14] and caspase 9 [16], respectively. Caspase-9 has a similar tetrapeptide motif in its NH_2_-terminus, so both compete for the BIR3 domain of XIAP [15]. Capase-3 is released by the interaction between NH_2_-terminus of Smac/DIABLO and BIR2 domain of XIAP [17].

### Smac/DIABLO sensitizes tumor cells to die by apoptosis

Several studies have shown that overexpression of Smac/DIABLO sensitizes neoplastic cells to apoptotic death [18,19]. These findings prompted the development of peptides derived from NH_2_-terminal of smac/DIABLO and small molecules that mimic Smac/DIABLO functions as therapeutic agents in order to induce death or to increase the apoptotic effect of chemotherapeutic agents. Permeable NH_2_-terminal peptides of Smac/DIABLO sensitize Hodgkin lymphoma cells to apoptosis mediated by B granzyme [18] and induce caspase-3 activation mediated by cytochrome- c [19]. Moreover, NH_2_-terminal peptides of Smac/DIABLO fused to Drosophila antennapaedia penetratin sequences enhance apoptosis mediated by different antineoplastic agents in breast cancer [20] and glioblastoma cell lines [21]. Similarly, a small Smac-mimic compound is able to increase the apoptotic effects of death factors such as TRAIL and TNF-α [22]. It is interesting to note that this small molecule induces apoptosis by itself in MDA-MB-231 breast cancer cells, which have high expression levels of XIAP and c-IAP1. In contrast, it only sensitizes MDA-MB-452 and T47D cells, which have low IAPs expression, to TRAIL or etoposide. [23]. Recently, it has been described that compounds that mimic Smac/DIABLO induce the activation of the NF-kB pathway eliciting TNF-α-dependent apoptosis via caspase-8 activation [24]. This activation depends on c-IAP1 and -2 degradation [25]. This was replicated *in vivo *using a malignant glioma xenograft mice model, in which co-administration of Smac/DIABLO peptides and TRAIL sensitized glioma cells to apoptotic death and induced tumor regression [26]. In the same line, tumor regression was observed when nude mice with xenografted hepatocarcinoma tumors where locally treated with an adenovirus expressing Smac/DIABLO and 5-Fluorouracyl [27]. Taken together, these results show that the NH_2_-terminal Smac/DIABLO derivatives and small molecules that mimic its function could be useful as adyuvant therapy in tumors with low levels of IAPs and as stand-alone therapy in tumors with high expression of IAPs. Further work is needed to address the potential use of these drugs in humans. Nevertheless, these studies show that particular expression levels of IAPs are key to the cellular response to these peptides or small molecules and underline the posible contribution of Smac/DIABLO levels to the intrinsic resistance to antineoplastic drugs.

### Smac/DIABLO and cancer progression

Due to the importance of Smac/DIABLO in determining the sensitivity of cancer cells to apoptotic death induced by diverse stimuli, it is important to investigate if its expression levels could be negatively regulated during the initiation or progression of cancer. This information could be useful as a prognostic or therapeutic marker or to provide new targets for drug design. As mentioned before, it could be expected that cells with lower Smac/DIABLO and therefore, higher apoptosis resistance, should be selected during cancer progression. This selection would contribute to the higher intrinsic resistance in more advanced cancer stages. All the studies have been summarized in Table 1.

**Table 1:** Expression of Smac/DIABLO in different tumors.

**Tumor**	**Smac/DIABLO expression**	**Molecule analyzed**	**Disease relationship.**	**Ref.**
RCC	**↓**	mRNA.	**Expression was low in patients with metastasis progress.**	[29]
	**↓**	Protein.	The levels of expression inversely correlate with grade disease.	[30]
	**-**	mRNA and protein.	**XIAP-Smac/DIABLO ratio increased from early to advance stages**.	[28]
Lung cancer	**↓**	mRNA.	Patients with low expression had worse prognosis.	[31]
TGCT	**↓**	mRNA.	**Smac/DIABLO expression diminished from normal testicular tissue to non-seminomatous germ cell tumours and stage III tumors.**	[34]
HCC	**↓**	mRNA and protein.	Tissues with HCC primary had low expression of Smac/DIABLO compared with normal organ.	[35]
CC	**↑**	mRNA.	**Smac/DIABLO expression is independent of stage. In a subset of tumors Smac/DIABLO is expressed *de novo***.	[36]
	**↑**	Protein.	Higher expression in adenocarcinoma than squamous tumors and correlates with a marker of angiogenesis. There was not any correlation with grade disease or survival rates.	[37]
GA	**↑**	mRNA.	**Smac/DIABLO expression was higher in gastric adenocarcinomas than in non-neoplastic gastric mucosa.**	[38]
Lymphomas (NHL and HL)	**-**	Protein.	There was not difference in Smac/DIABLO expression between clinically indolent and aggressive NHLs.	Ren. Y. et al.*
Carcinomas and sarcomas	**-**	Protein.	**Different tumors had distinct immunoreactivity against Smac/DIABLO.**	Yoo, N.J.et al**

*Y. Ren, N. Akyurek, E. Schlette, G.Z. Rassidakis, and L.J. Medeiros, Expression of Smac/DIABLO in B-cell non-Hodgkin and Hodgkin lymphomas. Hum Pathol 37 (2006) 1407-13., ** N.J. Yoo, H.S. Kim, S.Y. Kim, W.S. Park, C.H. Park, H.M. Jeon, E.S. Jung, J.Y. Lee, and S.H. Lee, Immunohistochemical analysis of Smac/DIABLO expression in human carcinomas and sarcomas. Apmis 111 (2003) 382-8.

Abbreviations: RCC, Renal Cell Carcinoma; NHL, non-Hodgkin lymphoma; HL, Hodgkin lymphoma; HCC, Hepatocellular carcinoma; CC, Cervical cancer; GA, Gastric adenocarcinoma; TGCT, Testicular germ cell tumors; XIAP, X-linked IAP.

### Inverse correlation between Smac/DIABLO expression and cancer progression

Two recent studies of Renal Cell Carcinoma (RCC) patients showed that Smac/DIABLO expression at both mRNA and protein level were not associated with stage or grade of tumor [28,29]. However, another study demonstrated a significative inverse correlation between Smac/DIABLO protein expression level and both stage and histologic grade of RCC [30]. Regardless of this discrepancy, a clear correlation with progression was made in the three studies: patients with metastasic disease had lower Smac/DIABLO levels than those with localized disease [28-30]. In addition, it was shown that patients that undergo radical nephrectomy for RCC with positive Smac/DIABLO expression had a longer disease-specific survival when compared with those with negative expression [30]. Moreover, RCC patients with low Smac/DIABLO expression had a four times higher risk to die than those with high expression [29].

Similarly, Sekimura et al have shown that Smac/DIABLO mRNA levels decreased significantly during lung cancer progression [31]. Since it has been shown that IAPs levels are upregulated in non small cell lung cancer [32], this reduction should increase further the apoptotic threshold of these cells. As expected from these results, decreasing XIAP expression *in vitro *sensitized lung cancer cells to apoptosis induced by diverse stimuli [33].

It has also been shown that testicular germ tumors with more advanced malignant phenotype presented lower Smac/DIABLO expression levels [34]. Moreover, the XIAP-Smac/DIABLO ratio increased significatively in clinical stage III tumors when compared with stages I or II. In this study an inverse correlation between Smac/DIABLO expression levels and cancer progression was also demonstrated [34].

Finally, it has also been shown that Smac/DIABLO mRNA and protein expression is reduced in hepatocellular carcinoma (HCC) when compared with normal hepatic tissue [35]. This reduction directly correlated with progression. Moreover, in this study, the authors showed that the IAP survivin, increased in parallel with cancer progression [35]. All these studies showed that Smac/DIABLO expression inversely correlated with cancer progression, aggressive behavior, as shown by metastasic disease, and bad prognosis. These correlations could be due to an increased apoptotic threshold, which should allow cancer cells to withstand not only invasion and metastasis, but to provide a relative increased intrinsic resistance to chemo- or radiotherapy. Further *in vivo *experiments specific for each tumor should help to solve this question.

### Direct correlation between Smac/DIABLO expression and cancer progression

Recently, our group analyzed the participation of Smac/DIABLO in cervical cancer patients. Surprisingly, we found that Smac/DIABLO expression did not correlate inversely with progression. As a matter of fact, normal cervix barely expressed the Smac/DIABLO RNA messenger. During cancer progression, a subset of cervical cancer samples expressed *de novo *this protein [36]. We could not find any correlation between Smac/DIABLO expression and stage or grade for both squamous cell carcinoma and adenocarcinoma [37]. However, higher immunostaining was directly associated with local recurrence in squamous cell carcinoma. We also established a positive correlation between Smac/DIABLO immunoreactivity and high microvascular density [37], which could be accounting for the recurrence results. More investigation is needed to clarify the relation between Smac/DIABLO and angiogenesis in cervical cancer.

A similar finding in gastric cancer patients supports these results [38]. Gastric adenocarcinomas showed a significant higher expression of Smac/DIABLO than non-neoplastic gastric mucosa [38]. However, no difference in the ratio XIAP-Smac/DIABLO between non-neoplasic gastric mucosa and gastric adenocarcinoma was found [38]. This discrepancy could be due to decrease in the expression of XAF1 (XIAP-associated factor-1) found in these samples [38]. XAF1 is a direct inhibitor of XIAP, so this decrease could be also be increasing the apoptotic threshold of these tumors [39]. Supporting these results, a recent report showed that a decrease of XIAP in gastric cancer cells increase the apoptotic response induced by cisplatin and mitomycin-c [40].

**Figure 1:**
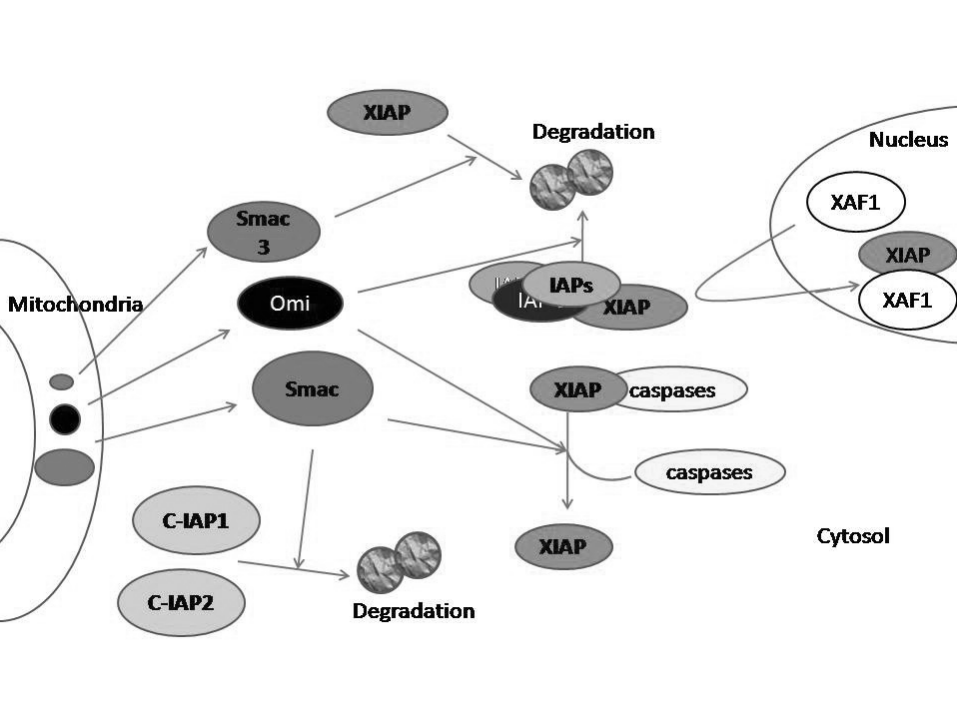
**Smac/DIABLO and other proteins are able to regulate the expression levels of inhibitors of apoptosis (IAPs)**. Smac/DIABLO-induced downregulation of c-IAP1 and c-IAP2 is mediated by ubiquitination and proteasomal degradation. Smac3, a Smac/DIABLO isoform generated by alternative splicing, induces the auto-ubiquitination and degradation of XIAP by the proteasome. Omi/HtrA2 degradates XIAP, c-IAP1 and c-IAP2. Another negative regulator of XIAP is XAF1.

**Figure 2:**
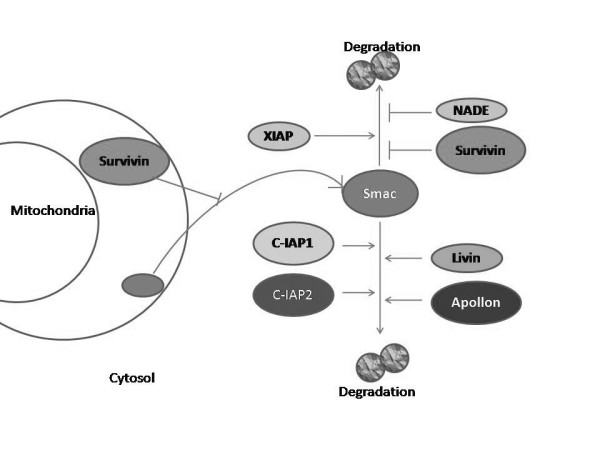
**The inhibitors of apoptosis (IAPs) regulate expression levels of Smac/DIABLO. Smac: Smac/DIABLO**. IAPs downregulate Smac/DIABLO by ubiquitination and proteasomal degradation. XIAP, c-IAP1 and c-IAP2 action is mediated by their RING domain. Smac/DIABLO degradation by XIAP is inhibited when the protein NADE is associated with them. Survivin inhibits the release of Smac/DIABLO from mitochondria after apoptotic stimuli. In addition, Survivin prevents Smac/DIABLO degradation in the cytosol.

Analyzing a single protein such as Smac/DIABLO or a particular IAP in cancer samples, will only give a partial vision of the mitochondrial apoptotic threshold, since it is known that these proteins are regulated by themselves and, in a tissue-specific pattern, specific IAPs functions overlap. In the case of IAPs (Figure 1), although Smac/DIABLO is not able to degradate XIAP, it induces the ubiquitination and degradation of other members of the family, such as c-IAP1 and c-IAP2 [41]. In addition, a recently cloned Smac/DIABLO isoform, Smac3, induces the auto-ubiquitination and degradation of XIAP [42]. As mentioned before, another negative regulator of XIAP is XAF1. This protein antagonizes the inhibitory activity of XIAP toward caspase-3 [39]. Similarly to IAPs, Smac/DIABLO is also regulated by IAPs family members (Figure 2). Livin is able to ubiquitinate and degrade Smac/DIABLO [43]. Also, in a reciprocal manner, XIAP, c-IAP1, c-IAP2 and Apollon induce the ubiquitination and degradation of Smac/DIABLO [11,44,45]. Although *in vivo *relevance has not yet been demonstrated for these mechanisms, a complicated scenario emerges from these studies, underlying the need for more research focusing not only in Smac/DIABLO protein and isoforms, but also in their interaction partners in a tissue-specific manner.

## Conclusion

The balance of IAPs and Smac/DIABLO dictates the apoptotic response to microenvironment clues (e.g. invasion and metastasis) and therapy. Due to this, it is important to perform protein expression studies, which are more informative than mRNA expression analyses, to survey not only Smac/DIABLO but also IAP family members to establish the relevance of these molecules in cancer progression and as possible prognostic markers. Although there is much to learn, we believe that directed therapy aimed to inhibit IAPs functions could be more efficient in those cancers with IAP overexpression or low expression of Smac/DIABLO. Nevertheless, caution is needed in those tumors in which the Smac/DIABLO and IAPs association with progression and/or prognosis is not clear, such as cervical and gastric cancer.

## Competing interests

The authors declare that they have no competing interests.

## Authors' contributions

GMR wrote the first draft, VM, GCC and JPRG contributed with specific sections, JM-Z reviewed the manuscript and wrote the final version. All authors read and approved the final manuscript.

## List of abbreviations

Smac/DIABLO: Second mitochondria-derived activator of caspase/direct inhibitor of apoptosis-binding protein with low pI; IAP: Inhibitor of Apoptosis Protein; TNF-α: Tumor necrosis factor-α; FADD: Fas Associated Death Domain; TRADD: TNF receptor associated-protein with death domain; DISC: Death Inducing Signal Complex; MOMP: Mitochondrial Outer Membrane Permeabilization; RING: Really Interesting New Gene; BIR: Baculovirus Inhibitor Repeat; MTS: Mitochondrial Targeting Signal; XIAP: X-linked inhibitor of apoptosis; AIF: Apoptosis inductor factor; cIAP1: cellular IAP-1; cIAP2: cellular IAP-2; Ts-IAP: Testis specific IAP; XAF1: XIAP-associated factor-1; RCC: Renal Cell Carcinoma; Omi: OmiHtrA2.
